# Supplementary material for: Molecular patterns and mechanisms of tumorigenesis in HPV-associated and HPV-independent sinonasal squamous cell carcinoma
Source: Nat Commun. 2025 Jun 11;16:5285. doi: 10.1038/s41467-025-59409-7 (PMC12159145; doi:10.1038/s41467-025-59409-7)
Supplement: Supplementary file 1 — Supplementary Information [file 41467_2025_59409_MOESM1_ESM.pdf]

Supplementary Information

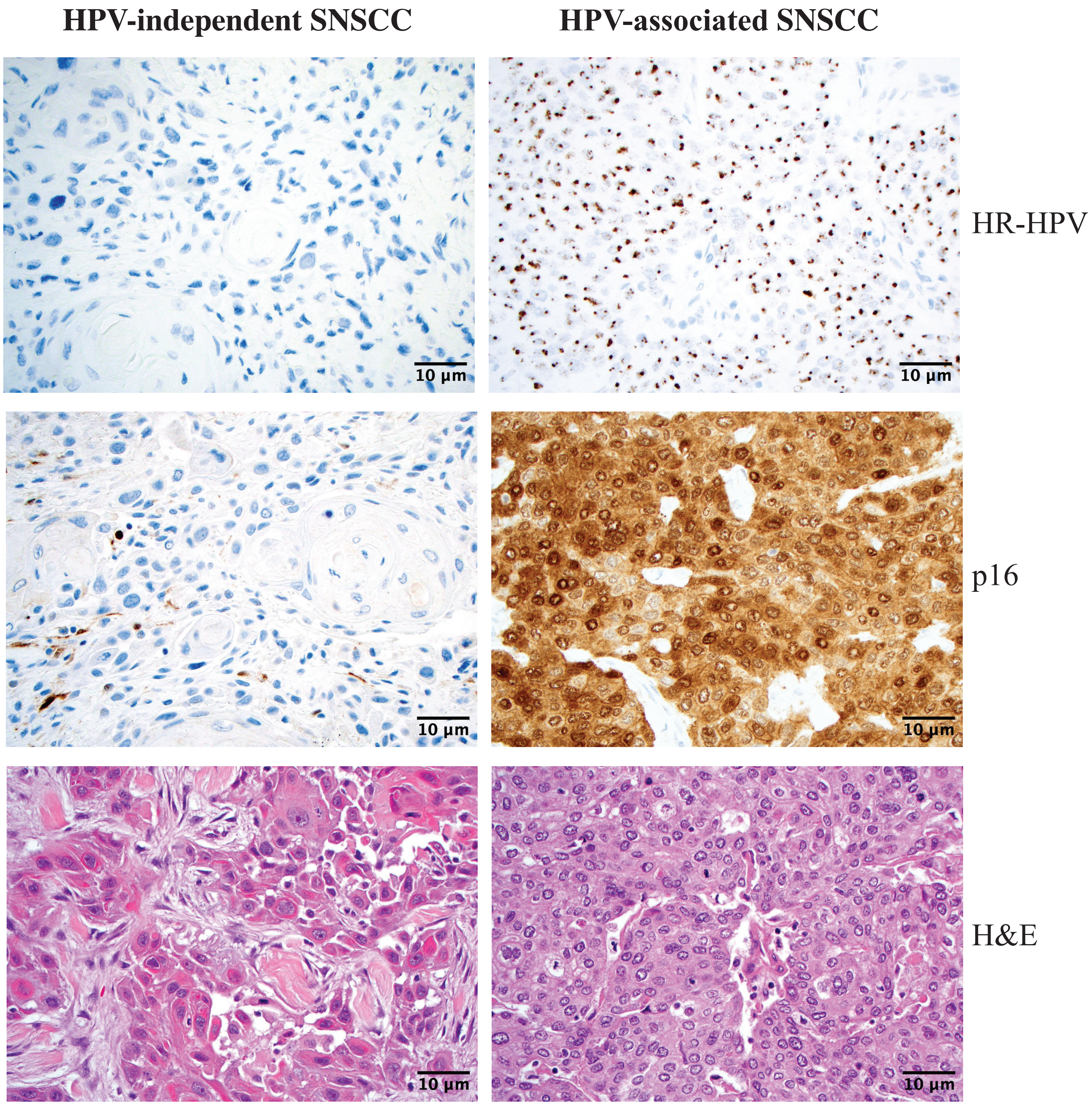

Supplementary Figure 1. Representative photomicrographs of HPV-independent (left column) and HPV-associated (right column) SNSCC. Rows correspond to different staining techniques: RNAscope HR-HPV in situ hybridization for high-risk HPV E6/E7 RNA (top row), p16 immunohistochemistry (middle row), and hematoxylin and eosin (H&E, bottom row). All images were captured at 40× magnification, and a scalebar of 10 µm is included in each panel.

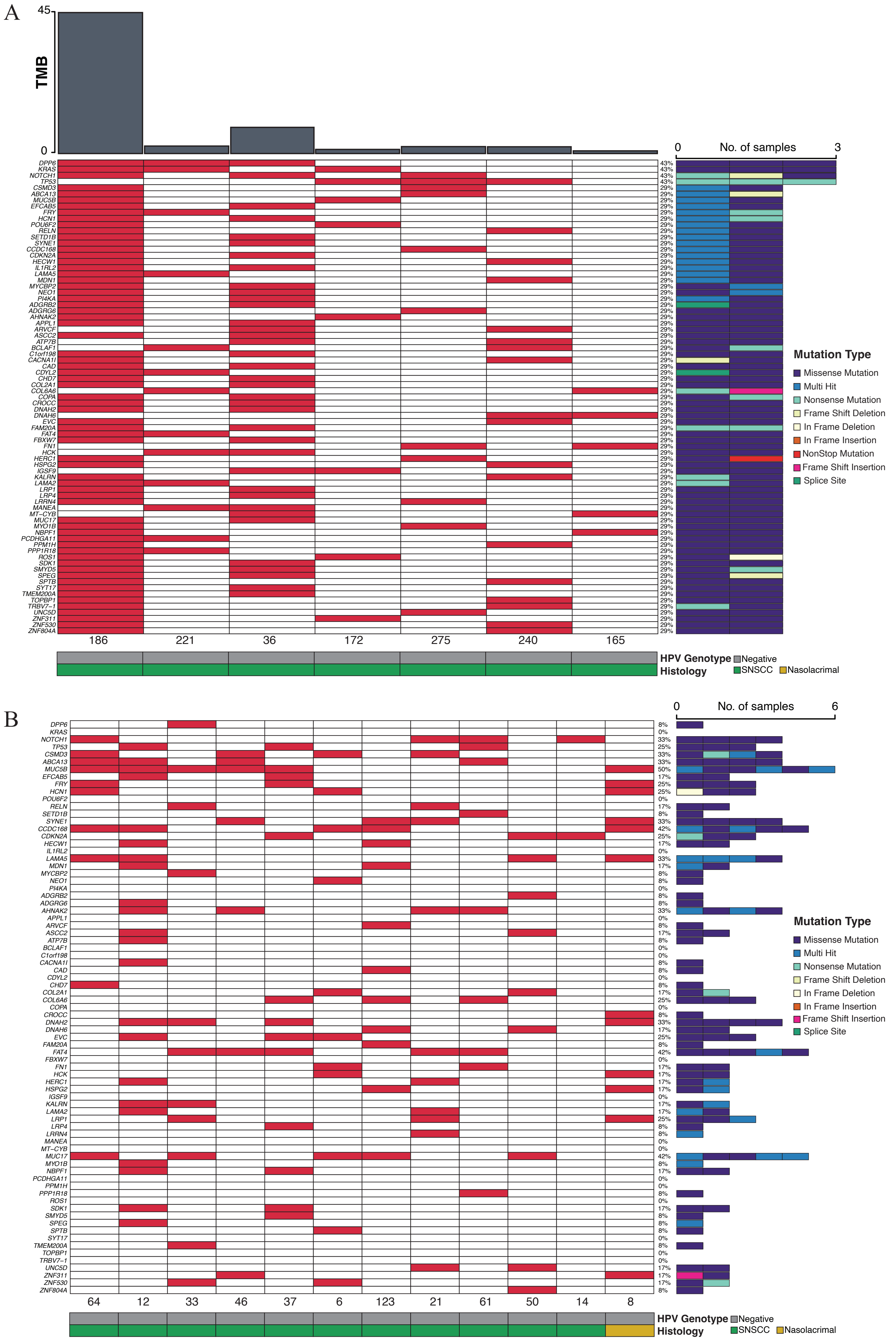

A
TMB
45
0
0
No. of samples
3
DPP6
KRAS
NOTCH1
TP53
CSMD3
ABCA13
MUC5B
EFCAB5
FRY
HCN1
POU6F2
RELN
SETD1B
SYNE1
CCDC168
CDKN2A
HECW1
IL1RL2
LAMA5
MDN1
MYCBP2
NEO1
PI4KA
ADGRB2
ADGRG6
AHNAK2
APPL1
ARVCF
ASCC2
ATP7B
BCLAF1
C1orf198
CACNA1I
CAD
CDYL2
CHD7
COL2A1
COL6A6
COPA
CROCC
DNAH2
DNAH6
EVC
FAM20A
FAT4
FBXW7
FN1
HCK
HERC1
HSPG2
IGSF9
KALRN
LAMA2
LRP1
LRP4
LRRN4
MANEA
MT-CYB
MUC17
MYO1B
NBPF1
PCDHGA11
PPM1H
PPP1R18
ROS1
SDK1
SMYD5
SPEG
SPTB
SYT17
TMEM200A
TOPBP1
TRBV7-1
UNC5D
ZNF311
ZNF530
ZNF804A
186
221
36
172
275
240
165
Mutation Type
Missense Mutation
Multi Hit
Nonsense Mutation
Frame Shift Deletion
In Frame Deletion
In Frame Insertion
NonStop Mutation
Frame Shift Insertion
Splice Site
HPV Genotype
Negative
Histology
SNSCC
Nasolacrimal
B
0
No. of samples
6
64
12
33
46
37
6
123
21
61
50
14
8
Mutation Type
Missense Mutation
Multi Hit
Nonsense Mutation
Frame Shift Deletion
In Frame Deletion
In Frame Insertion
Frame Shift Insertion
Splice Site
HPV Genotype
Negative
Histology
SNSCC
Nasolacrimal

Supplementary Figure 2. High-throughput sequencing of HPV-independent SNSCC reveals distinct mutational patterns. (A) Whole-exome sequencing was performed in HPV-independent SNSCC with matched normal DNA (n=7) and somatic variants assessed using a panel of normal genomes. Mutations in all genes are represented. **(B)** Whole-exome sequencing was performed in HPV-independent SNSCC without matched normal DNA (n=12) and somatic variants assessed using a panel of normal genomes. Mutations in all genes are represented. Source data are provided as a **Source data** file.

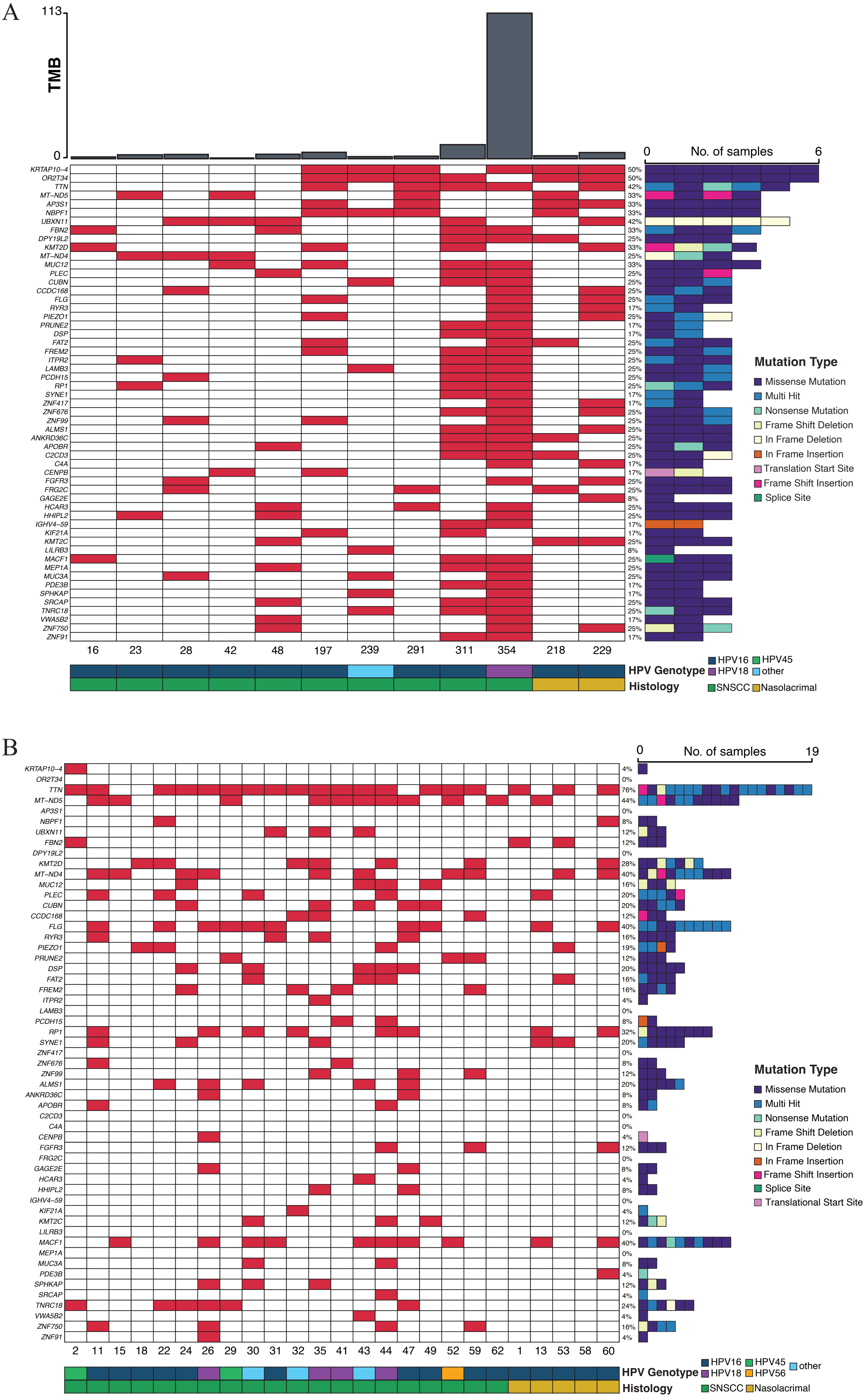
A
TMB
113
0
No. of samples
0
6
KRTAP10−4 50%
OR2T34 50%
TTN 42%
MT−ND5 33%
AP3S1 33%
NBPF1 33%
UBXN11 42%
FBN2 33%
DPY19L2 25%
KMT2D 33%
MT−ND4 25%
MUC12 33%
PLEC 25%
CUBN 25%
CCDC168 25%
FLG 25%
RYR3 17%
PIEZO1 25%
PRUNE2 17%
DSP 17%
FAT2 25%
FREM2 25%
ITPR2 25%
LAMB3 25%
PCDH15 25%
RP1 25%
SYNE1 17%
ZNF417 17%
ZNF676 25%
ZNF99 25%
ALMS1 25%
ANKRD36C 25%
APOBR 25%
C2CD3 25%
C4A 17%
CENPB 17%
FGFR3 25%
FRG2C 25%
GAGE2E 8%
HCAR3 25%
HHIPL2 25%
IGHV4−59 17%
KIF21A 17%
KMT2C 25%
LILRB3 8%
MACF1 25%
MEP1A 25%
MUC3A 25%
PDE3B 17%
SPHKAP 17%
SRCAP 25%
TNRC18 25%
VWA5B2 17%
ZNF750 25%
ZNF91 17%
16 23 28 42 48 197 239 291 311 354 218 229
HPV Genotype
Histology
HPV16 HPV45 HPV18 other
SNSCC Nasolacrimal
Mutation Type
Missense Mutation
Multi Hit
Nonsense Mutation
Frame Shift Deletion
In Frame Deletion
In Frame Insertion
Translation Start Site
Frame Shift Insertion
Splice Site
B
No. of samples
0
19
KRTAP10−4 4%
OR2T34 0%
TTN 76%
MT−ND5 44%
AP3S1 0%
NBPF1 8%
UBXN11 12%
FBN2 12%
DPY19L2 0%
KMT2D 28%
MT−ND4 40%
MUC12 16%
PLEC 20%
CUBN 20%
CCDC168 12%
FLG 40%
RYR3 16%
PIEZO1 19%
PRUNE2 12%
DSP 20%
FAT2 16%
FREM2 16%
ITPR2 4%
LAMB3 0%
PCDH15 8%
RP1 32%
SYNE1 20%
ZNF417 0%
ZNF676 8%
ZNF99 12%
ALMS1 20%
ANKRD36C 8%
APOBR 8%
C2CD3 0%
C4A 0%
CENPB 4%
FGFR3 12%
FRG2C 0%
GAGE2E 8%
HCAR3 4%
HHIPL2 8%
IGHV4−59 0%
KIF21A 4%
KMT2C 12%
LILRB3 0%
MACF1 40%
MEP1A 0%
MUC3A 8%
PDE3B 4%
SPHKAP 12%
SRCAP 4%
TNRC18 24%
VWA5B2 4%
ZNF750 16%
ZNF91 4%
2 11 15 18 22 24 26 29 30 31 32 35 41 43 44 47 49 52 59 62 1 13 53 58 60
HPV Genotype
Histology
HPV16 HPV45 HPV18 HPV56 other
SNSCC Nasolacrimal
Mutation Type
Missense Mutation
Multi Hit
Nonsense Mutation
Frame Shift Deletion
In Frame Deletion
In Frame Insertion
Frame Shift Insertion
Splice Site
Translational Start Site

Supplementary Figure 3. High-throughput sequencing of HPV-associated SNSCC reveals distinct mutational patterns. **(A)** Whole-genome sequencing was performed in HPV-associated SNSCC with matched normal DNA (n=12) and somatic variants were assessed. Mutations in all genes are represented. **(B)** Whole-genome sequencing was performed in HPV-associated SNSCC without matched normal DNA (n=25) and somatic variants assessed using a panel of normal genomes. Mutations in all genes are represented. Source data are provided as a **Source data** file.

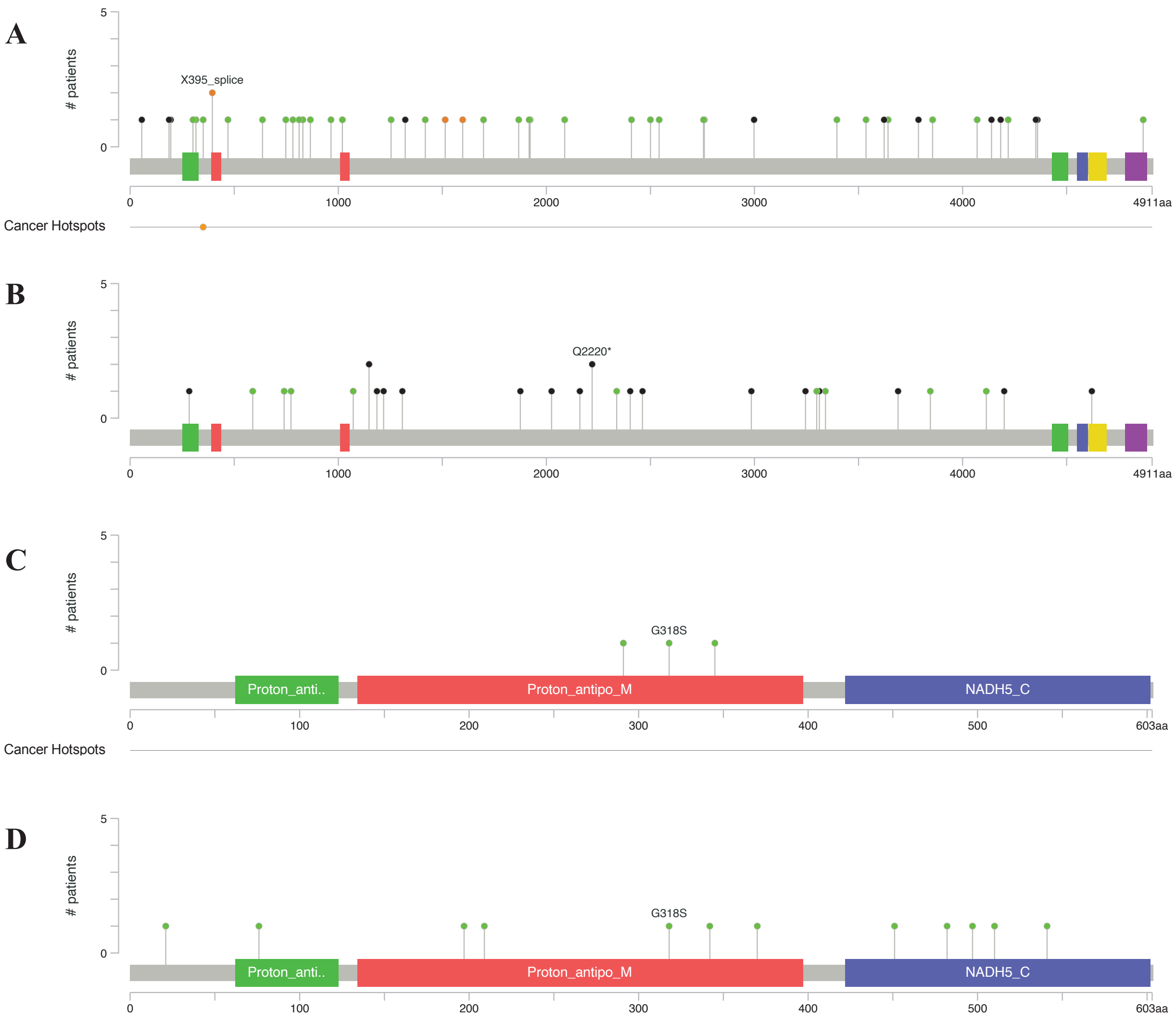

Supplementary Figure 4. HNSCC and CSCC lack *KMT2C* N729D mutations and *MT-ND5* frameshift insertion/deletions at c.891-892 or c.1278-1279. (A) TCGA was evaluated for *KMT2C* mutations in HNSCC and 0/46 N729D mutations were observed. **(B)** TCGA was evaluated for *KMT2C* mutations in CSCC and 0/32 N729 mutations were observed. **(C)** TCGA was evaluated for *MT-ND5* mutations in HNSCC and 0/3 *MT-ND5* c.891-892 or c.1278-1279 frameshift insertion/deletions were noted. **(D)** TCGA was evaluated for *MT-ND5* mutations in CSCC and 0/12 *MT-ND5* c.891-892 or c.1278-1279 frameshift insertion/deletions were noted. Source data are provided as a **Source data** file.

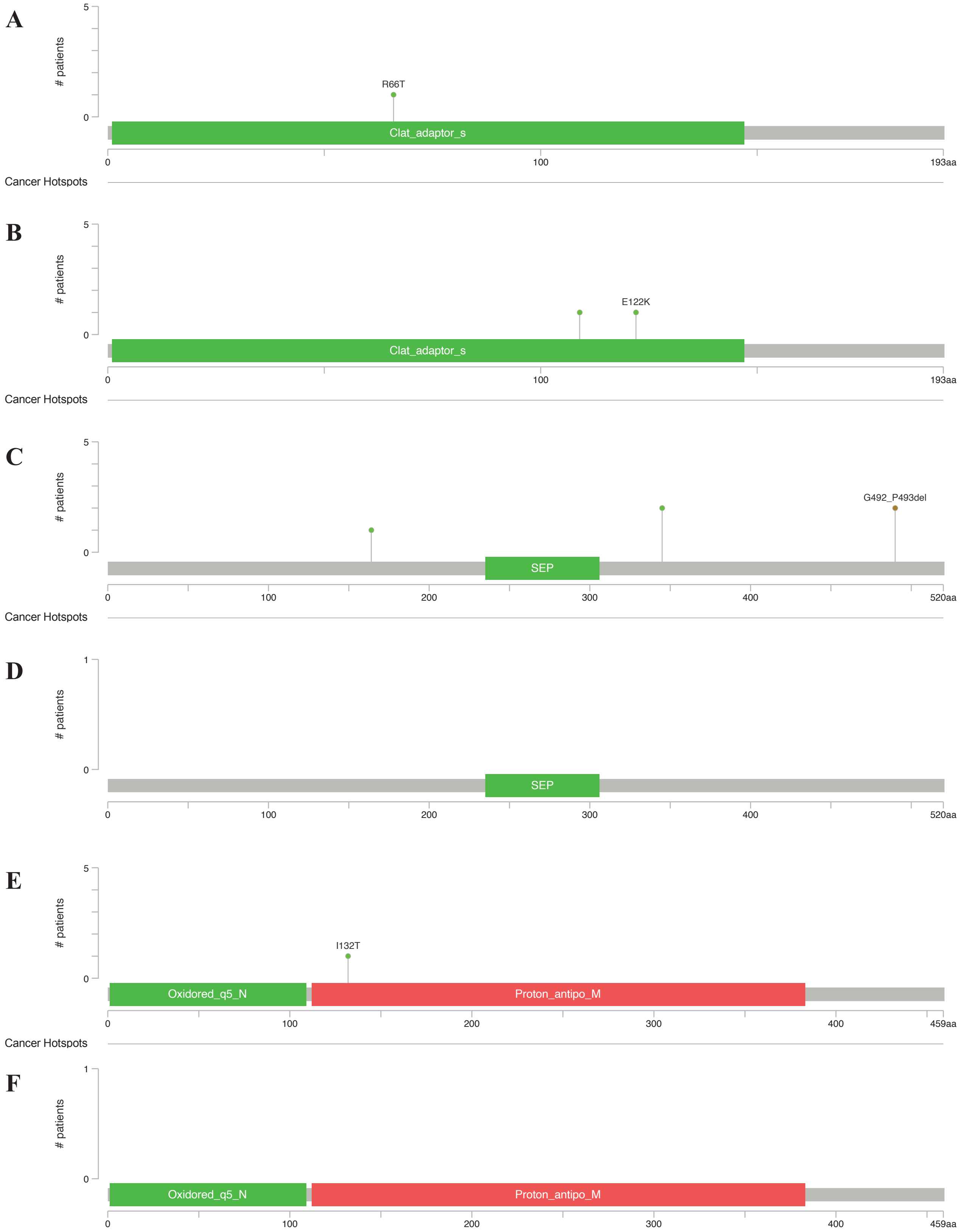

Supplementary Figure 5. HNSCC and CSCC either lack or have few mutations in *AP3S1*, *UBXN11*, and *MT-ND4*. TCGA was evaluated for *AP3S1* mutations in HNSCC **(A)** and CSCC **(B)** and few mutations were observed. TCGA was evaluated for *UBXN11* mutations in HNSCC **(C)** and CSCC **(D)** and few mutations were observed. TCGA was evaluated for *MT-ND4* mutations in HNSCC **(E)** and CSCC **(F)** and few mutations were observed. None of the recurrent changes in these genes were seen as in HPV-associated SNSCC. Source data are provided as a **Source data** file.

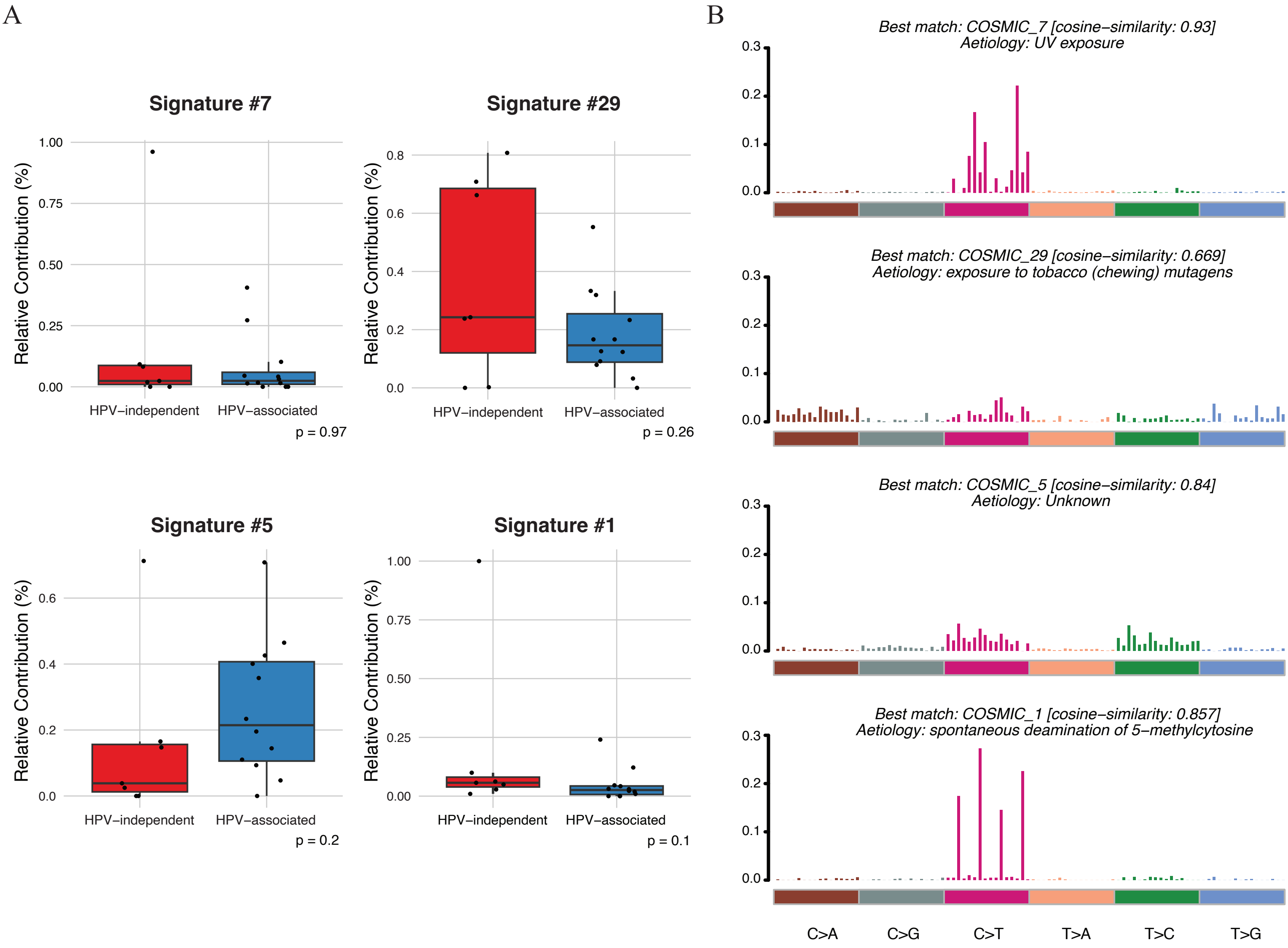

Supplementary Figure 6. Additional pathway enrichment in HPV-independent and HPV-associated SNSCC. **(A)** Additional dox plot comparisons of pathway enrichment in HPV-independent and HPV-associated SNSCC. **(B)** Signature profiles in the HPV-associated SNSCC cohort. Source data are provided as a **Source data** file.

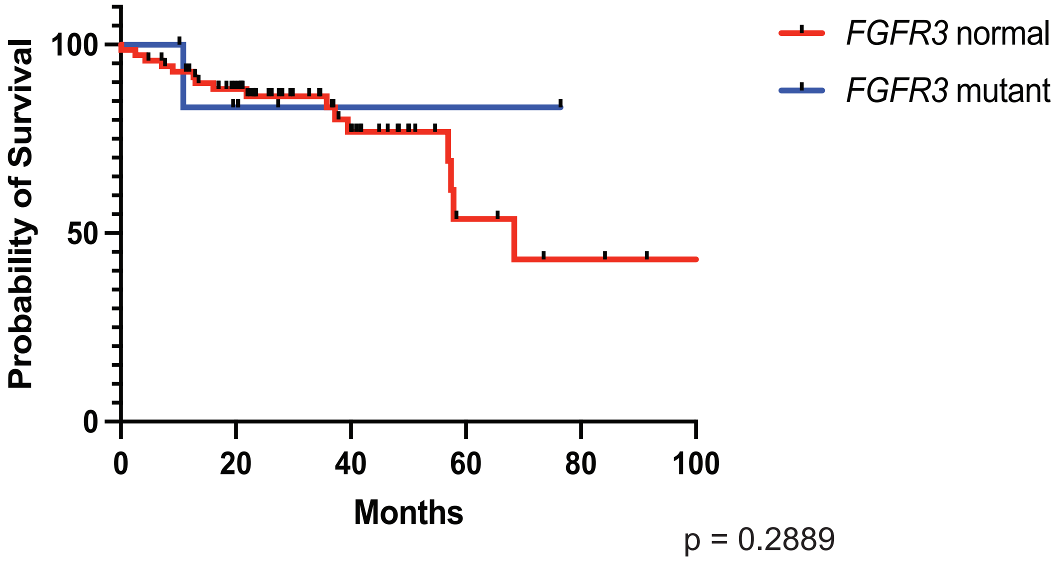

Supplementary Figure 7. No association with overall survival and *FGFR3* mutation status in HNSCC. (A) HPV-associated HNSCC from TCGA with *FGFR3* mutations and overall survival (P = 0.2889). Source data are provided as a **Source data** file.

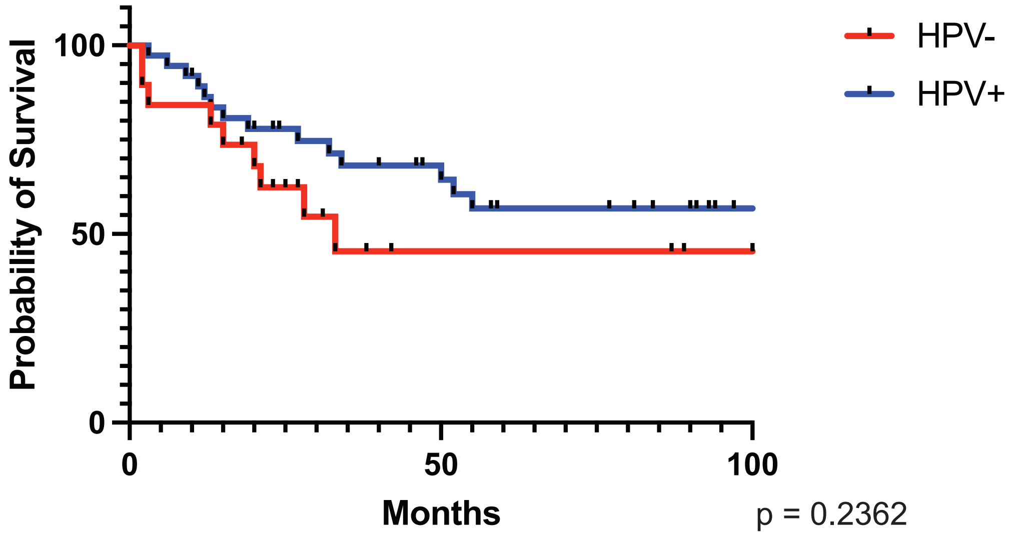

Supplementary Figure 8. Survival Analysis of HPV-Associated and HPV-Independent SNSCC. HPV-associated SNSCC showed a trend toward improved survival, but this was not statistically significant (*P* = 0.2362). Source data are provided as a **Source data** file.

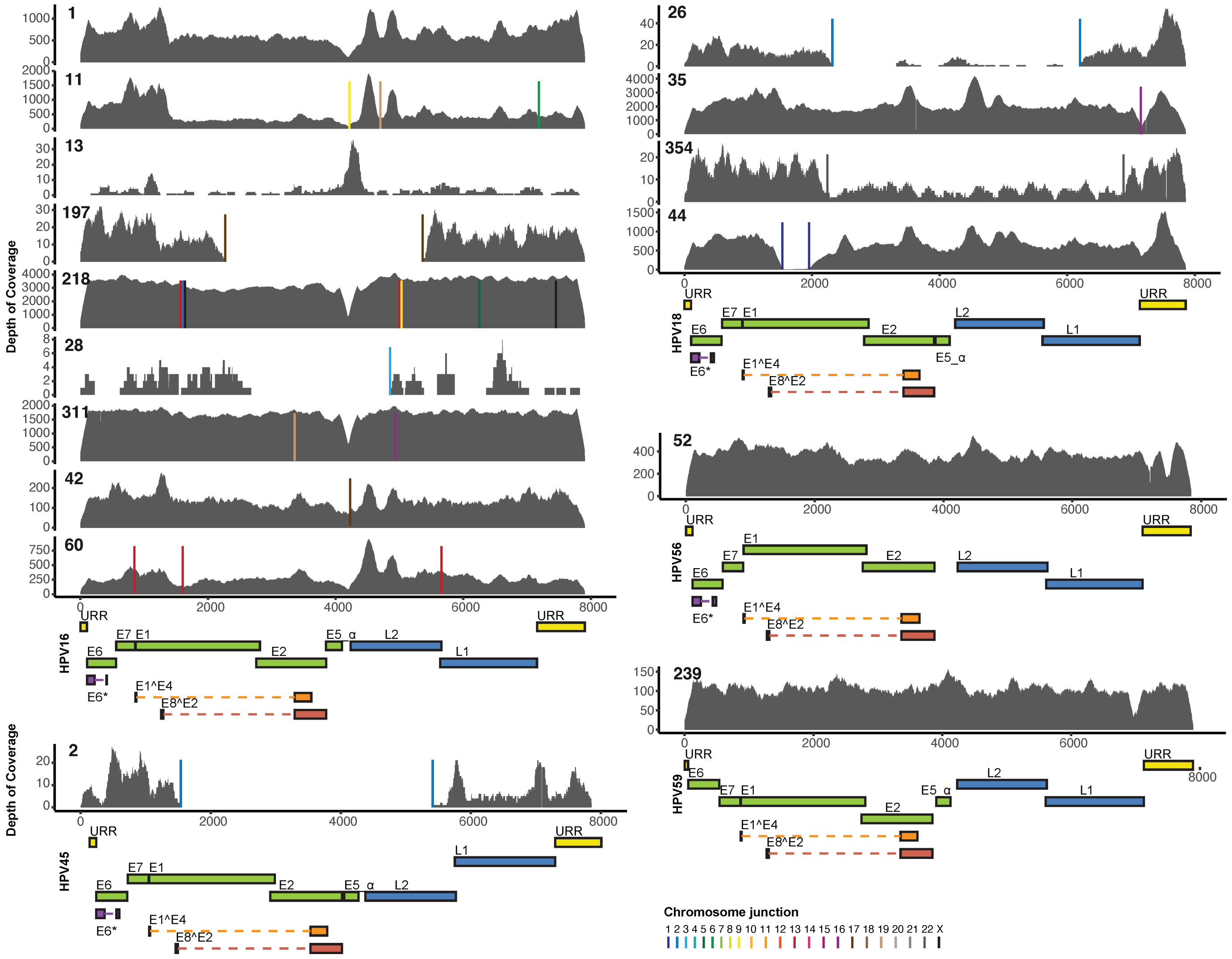

Supplementary Figure 9. Analysis of viral integration breakpoints in HPV-associated SNSCC. Distribution of breakpoints across HPV genomes detected in HPV-associated SNSCC samples. For each HPV type, the upper panels display the depth of viral genome coverage, while colored vertical lines indicate detected integration breakpoints along the viral genome. The lower panels outline the genomic structure of the HPV genome, including annotated regions (e.g., E1, E2, E5, E6, E7, L1, L2, α and URR). Chromosome junctions are represented by colored bars corresponding to chromosomal locations, as shown in the legend at the bottom. Source data are provided as a **Source data** file.

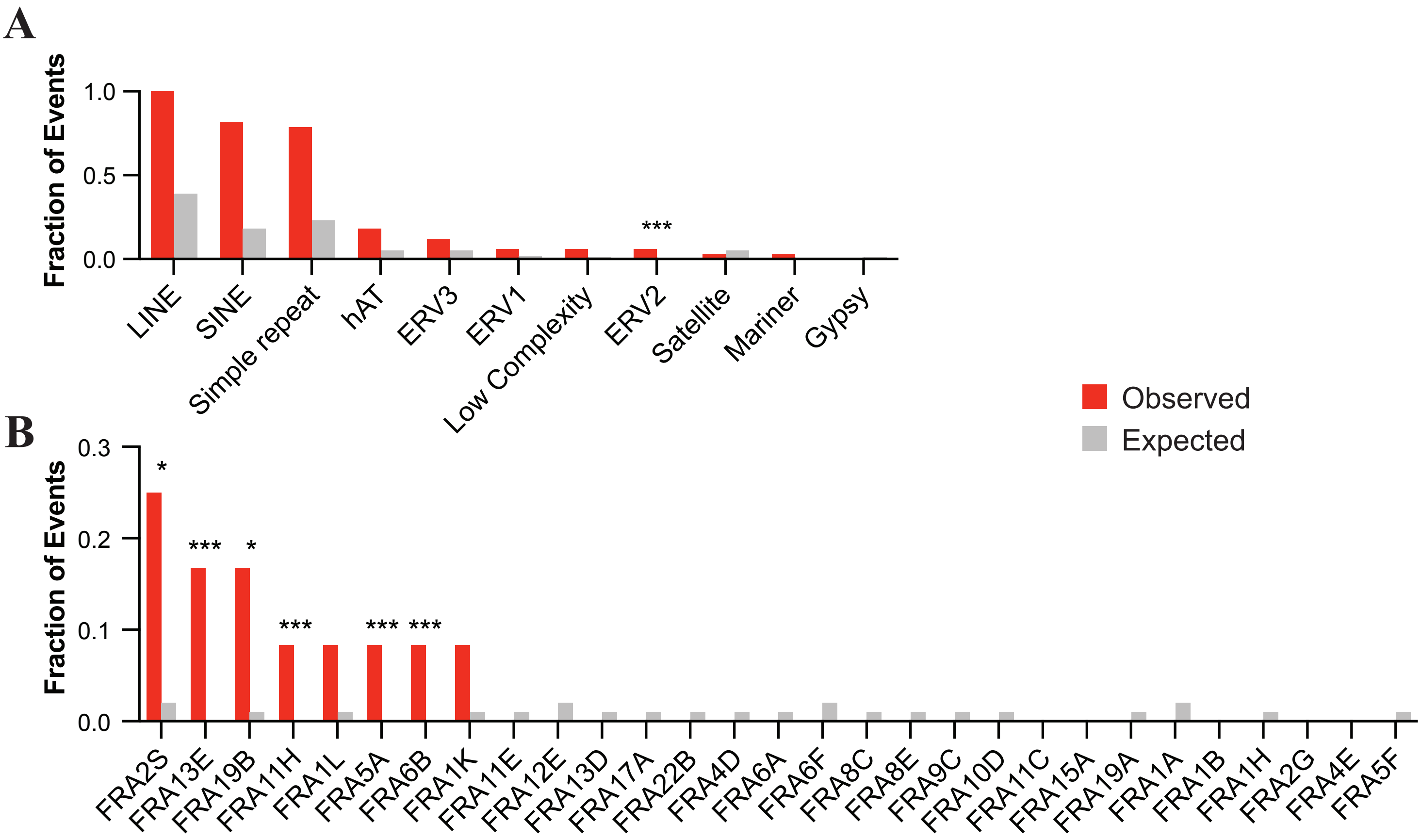

Supplementary Figure 10. Enrichment of integration events across repetitive elements and fragile sites in HPV-associated SNSCC. (A) Observed (red) versus expected (gray) integration events across repetitive elements, with significant enrichment in SINE, LINE, and simple repeats (*******p < 0.001). **(B)** Integration events at fragile sites, with all 27 events overlapping fragile sites. Significant enrichment is seen in FRA2S, FRA13E, FRA19B, FRA1H, FRA1L, and FRA6B (*****p < 0.05, *******p < 0.001). Source data are provided as a **Source data** file.

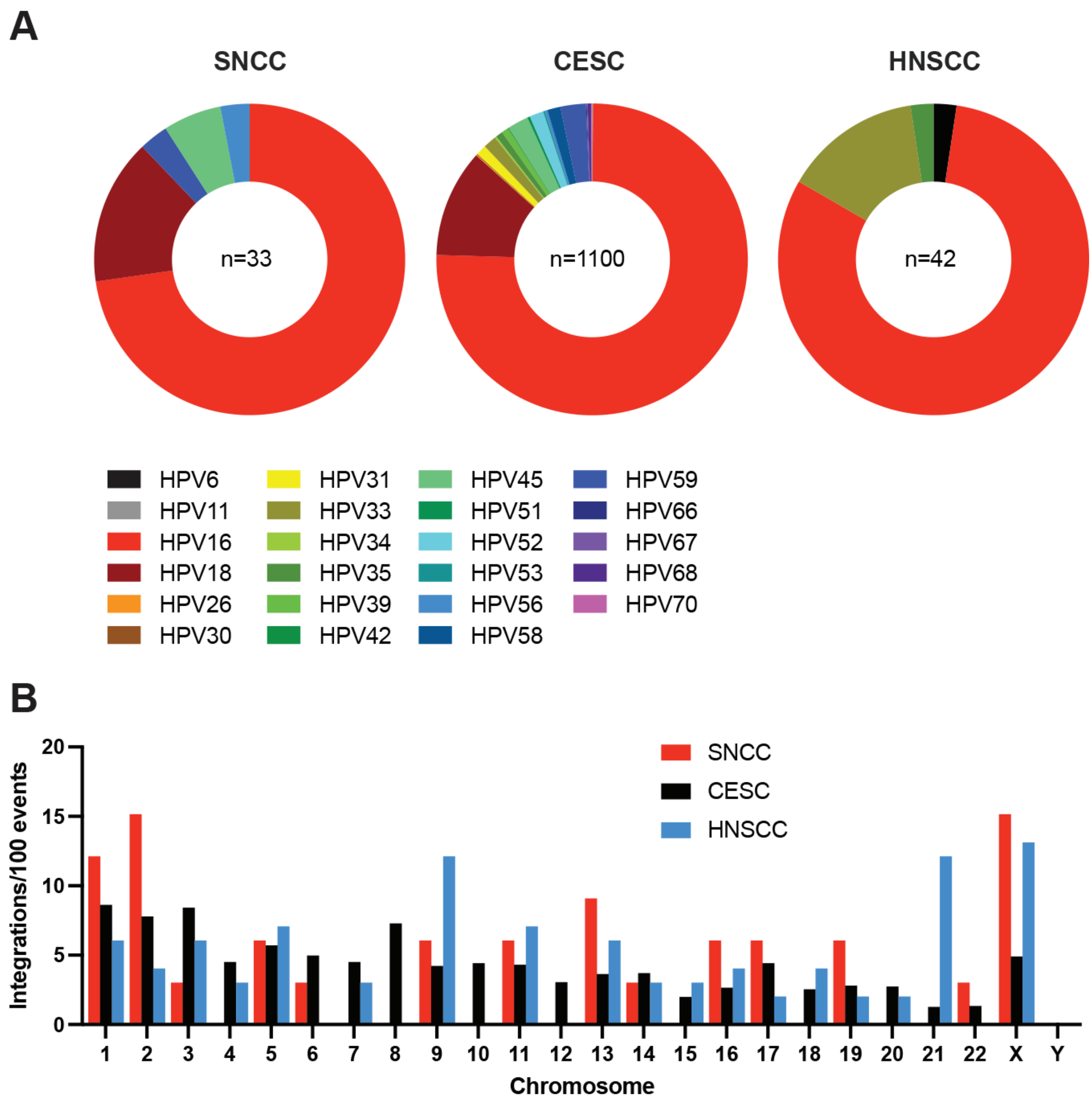

Supplementary Figure 11. HPV genotype distribution and integration frequencies in SNCC, CESC, and HNSCC. (A) Distribution of HPV genotypes in SNCC (n=33), CESC (n=1100), and HNSCC (n=42). Donut charts illustrate the proportional representation of HPV genotypes, with HPV16 (red) the most prevalent across all cancer types. The legend identifies HPV types corresponding to each color. **(B)** Distribution of integration frequencies across chromosomes in SNCC, CESC, and HNSCC. Integration events per 100 are shown for each chromosome. Similarities in integration event distribution are noted across the three cancer types. Source data are provided as a **Source data** file.

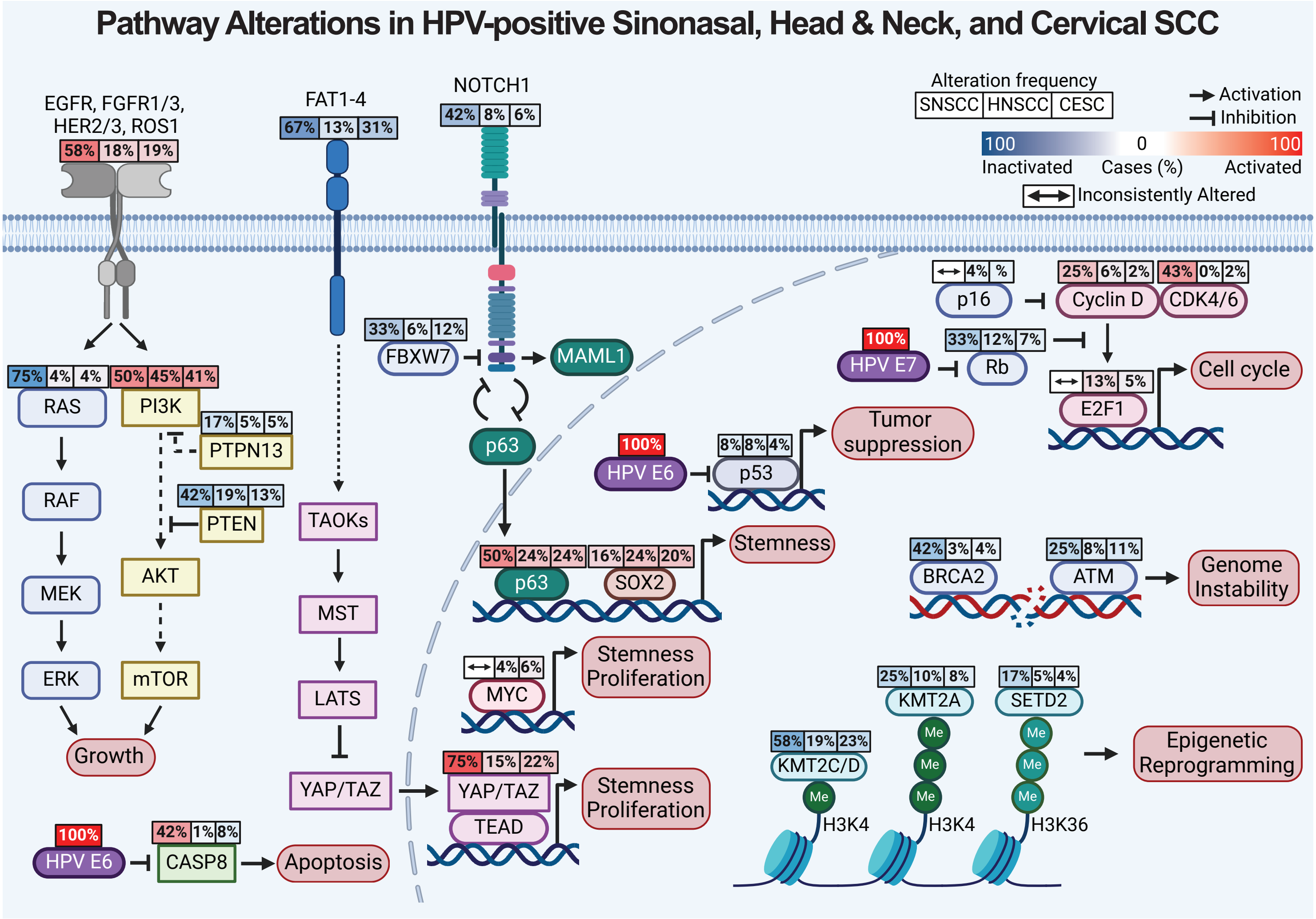

Supplementary Figure 12. Pathway alteration comparison of HPV-associated disease. Incorporation of mutation and copy number alteration analysis reveals targetable pathways enhanced in HPV-associated SNSCC (left), compared to HPV-associated HNSCC (middle), and HPV-associated CESC (right). Created in BioRender. Team, S. (2025) https://BioRender.com/j76q749. Source data are provided as a **Source data** file.

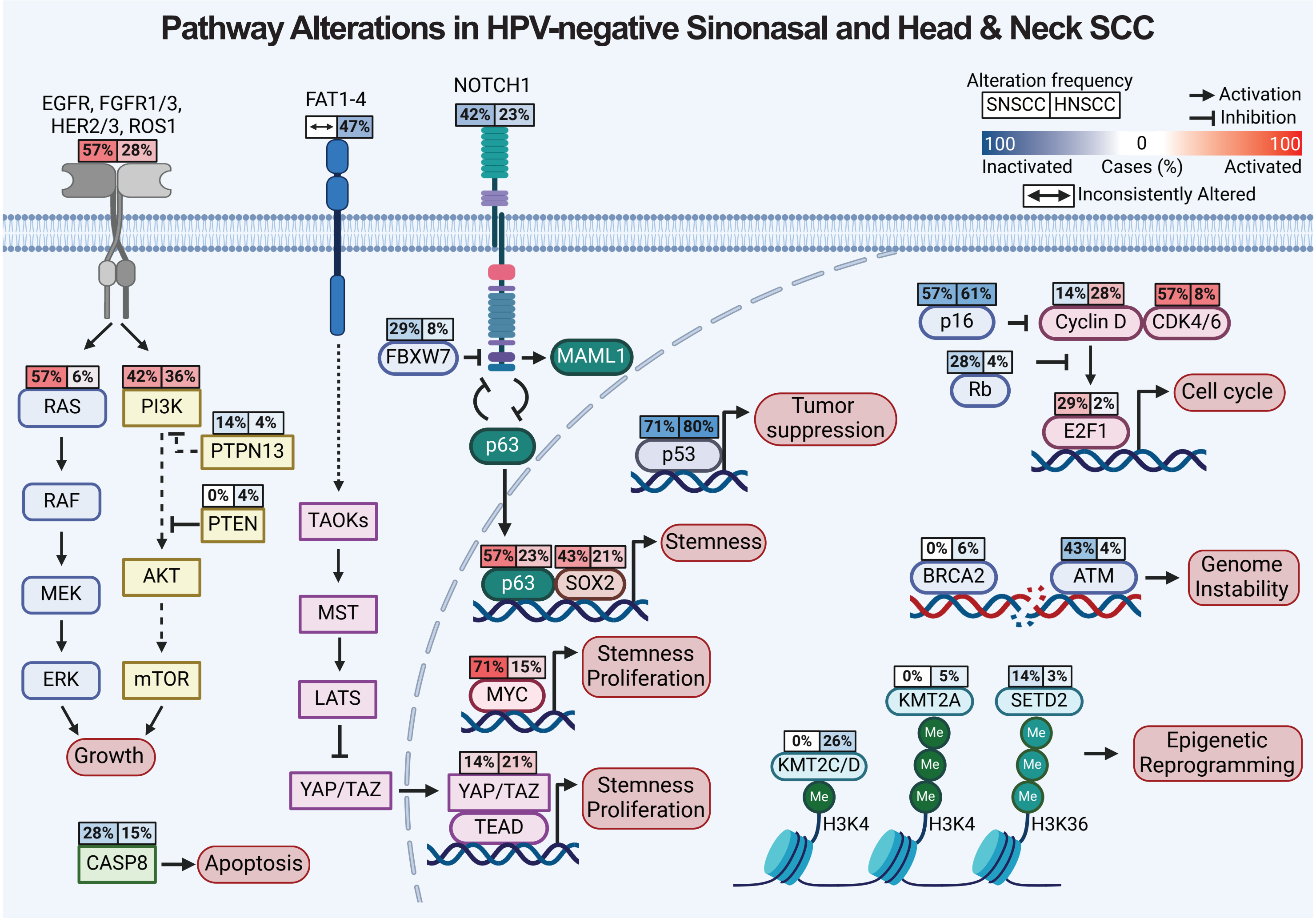

Supplementary Figure 13. Pathway alteration comparison of HPV-independent disease. Incorporation of mutation and copy number alteration analysis reveals targetable pathways enhanced in HPV-independent SNSCC (left) compared to HPV-independent HNSCC (right). Created in BioRender. Team, S. (2025) https://BioRender.com/e33g638. Source data are provided as a **Source data** file.

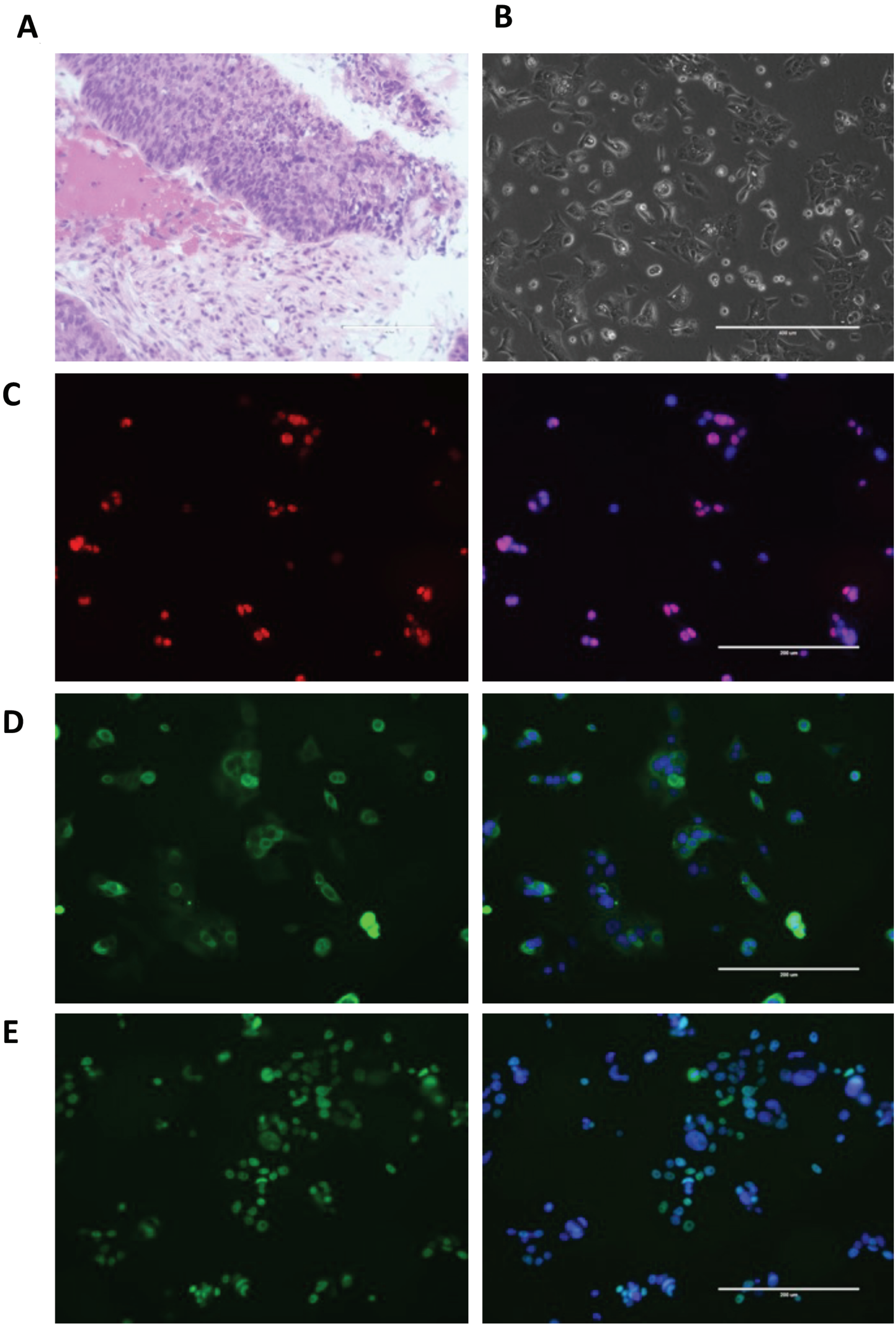

Supplementary Figure 14. Validation of HPV-associated SNSCC cell line. (A) H&E staining of original primary tumor. **(B)** Bright field image and Immunofluorescence staining for **(C)** P63, **(D)** cytokeratin AE1/AE3, and **(E)** P40 of cell line established from primary tumor. Scale bar in A and B are 400 μm and 200 μm in C-E.
