# Supplementary material for: Molecular patterns and mechanisms of tumorigenesis in HPV-associated and HPV-independent sinonasal squamous cell carcinoma
Source: Nat Commun. 2025 Jun 11;16:5285. doi: 10.1038/s41467-025-59409-7 (PMC12159145; doi:10.1038/s41467-025-59409-7)
Supplement: Supplementary file 2 — Description of Additional Supplementary Files [file 41467_2025_59409_MOESM2_ESM.pdf]

Description of Additional Supplementary Files

Supplementary Data 1. SNSCC mutations in genes of interest in HPV-independent samples with matched normals.

Supplementary Data 2. Mutations in genes of interest in HPV-independent SNSCC samples with a panel of normal genomes.

Supplementary Data 3. Mutations in genes of interest in HPV-associated SNSCC samples with matched normal DNA.

Supplementary Data 4. Mutations in genes of interest in HPV-associated SNSCC samples with a panel of normal genomes.

Supplementary Data 5. Summary of HPV integration events.

Supplementary Data 6. Summary of sequencing metrics and average coverage for all tumor and normal samples analyzed using whole-exome sequencing (WES) or whole-genome sequencing (WGS).
