# Supplementary material for: Molecular patterns and mechanisms of tumorigenesis in HPV-associated and HPV-independent sinonasal squamous cell carcinoma
Source: Nat Commun. 2025 Jun 11;16:5285. doi: 10.1038/s41467-025-59409-7 (PMC12159145; doi:10.1038/s41467-025-59409-7)
Supplement: Supplementary file 9 — Reporting Summary [file 41467_2025_59409_MOESM9_ESM.pdf]

nature portfolio

Corresponding author(s): Nyall R. London Jr.

Last updated by author(s): Apr 15, 2025

Reporting Summary

Nature Portfolio wishes to improve the reproducibility of the work that we publish. This form provides structure for consistency and transparency in reporting. For further information on Nature Portfolio policies, see our Editorial Policies and the Editorial Policy Checklist.

Statistics

For all statistical analyses, confirm that the following items are present in the figure legend, table legend, main text, or Methods section.

n/a	Confirmed	
☐	☒	The exact sample size (*n*) for each experimental group/condition, given as a discrete number and unit of measurement
☐	☒	A statement on whether measurements were taken from distinct samples or whether the same sample was measured repeatedly
☐	☒	The statistical test(s) used AND whether they are one- or two-sided<br>*Only common tests should be described solely by name; describe more complex techniques in the Methods section.*
☐	☒	A description of all covariates tested
☐	☒	A description of any assumptions or corrections, such as tests of normality and adjustment for multiple comparisons
☐	☒	A full description of the statistical parameters including central tendency (e.g. means) or other basic estimates (e.g. regression coefficient) AND variation (e.g. standard deviation) or associated estimates of uncertainty (e.g. confidence intervals)
☐	☒	For null hypothesis testing, the test statistic (e.g. *F*, *t*, *r*) with confidence intervals, effect sizes, degrees of freedom and *P* value noted<br>*Give P values as exact values whenever suitable.*
☒	☐	For Bayesian analysis, information on the choice of priors and Markov chain Monte Carlo settings
☒	☐	For hierarchical and complex designs, identification of the appropriate level for tests and full reporting of outcomes
☒	☐	Estimates of effect sizes (e.g. Cohen's *d*, Pearson's *r*), indicating how they were calculated

Our web collection on statistics for biologists contains articles on many of the points above.

Software and code

Policy information about availability of computer code

Data collection	For mutation frequency analyses, The Cancer Genome Atlas (TCGA) HNSCC and CESC datasets were extracted using cBioportal.
Data analysis	Bioinformatics Analyses: Sequencing data were demultiplexed using Illumina bcl2fastq2 (v.2.17.1.14) with default filters. Trimgalore (v0.6.7) was used to trim off adaptor sequences and low-quality bases. Reads were then aligned against the hg38 genome using BWA-MEM (v0.7.17, Sentieon 202010.02 release). Duplicate reads were removed using Picard tools (v2.9.0, Sentieon 202010.02 release). Final recalibrated alignment files were created using Genome Analysis Toolkit (GATK, v3.8.0, Sentieon 202010.02 release). To determine coverage at different levels of partitioning and aggregation, Samtools depth v1.10 & GATK DepthOfCoverage v3.8.0 were used for WES data, while Bedtools genomecov v2.30.0 was used for WGS data. Somatic variants between the tumor-normal pairs were called using GATK MuTect2 (v3.8.0, Sentieon 202010.02 release). For samples without matched normal, the panel of normal genomes from the 1000 Genomes database provided by GATK was used. GATK HaplotypeCaller (v3.8.0, Sentieon 202010.02 release) was used to call germline variants in each sample. Passed somatic and germline variants were converted into Mutation Annotation Format files using vcf2maf (v1.6.19), and then summarized and visualized using maftools (v2.10.05). Copy number analyses were performed using CNVkit (v0.9.4) for samples with matched normal, or Control-FREEC (v11.6) for samples without matched normal. Mutational signature analysis was performed using maftools (v2.18.0). The relative signature contribution barplot and boxplots were created using ggplot (v2_3.5.0). The mean comparison p-values were added to the boxplots using ggpubr (v0.6.0.999).<br>For mutation frequency analyses, The Cancer Genome Atlas (TCGA) HNSCC and CESC datasets were extracted using cBioportal. HNSCC tumor HPV status was based on RNAseq reads aligning to the HPV genome, yielding 449 HPV-negative and 78 HPV-positive HNSCC tumors of all anatomic sites For CESC tumors, only cervical squamous cell carcinoma (n=141) were included. Cervical adenocarcinoma and cervical adenosquamous carcinoma as well as HPV-negative cervical tumors were excluded. Cancer-associated signaling pathways were prioritized based on pathway constituent protein products of genes frequently altered in SNSCC, HNSCC, or CESC. Genomic alteration frequency was defined as the proportion of samples with either somatic coding sequence alteration (single nucleotide alteration or short insertion/deletion)

or copy number alteration (log2 copy number gain/loss >0.3). Pathway figures were drawn using BioRender (biorender.com).
HPV Integration Analysis: HPV integration analysis was conducted by first extracting the reads not mapping to the human genome or those with one unmatched mate-pair read. These reads were adapter and quality trimmed using fastp (0.23.2) remapped to a reference genome containing hg38 and all HPV genomes in the Papillomavirus Episteme (PaVE) as of 2018. From these alignments, the methods used in oncovirus_tools (https://github.com/gstarrett/oncovirus_tools, https://doi.org/10.5281/zenodo.3661416) were modified to determine integration sites for all detected HPV genomes. Integration sites were annotated using bedtools and the coordinates for all hg38 genes, fragile sites (https://webs.iiitd.edu.in/raghava/humcfs/), and repeatMasker elements. HPV types were called based on alignment and assembly data. For a type to be called, tumor sequencing must have had at least 10% of the HPV type genome covered with 2x or higher read depth. Concordance was checked against the de novo assemblies, which were matched to HPV types using blast taking the lowest e-value match (maximum cutoff of 1e-10) and the HPV type. Integration site enrichment near repeat elements and fragile sites was calculated by generating 1000 random integration events on the mappable hg38 genome. Chi-square calculation of observed versus expected integrations near repeat elements and fragile sites and were Bonferroni corrected for multiple comparisons. For integration site distribution and HPV type comparison across SNSCC, HNSCC, and CESC, data were retrieved and from (http://www.vis-atlas.tech/) and cell lines and precancerous lesions were excluded.
Statistical Analysis: For comparisons between two groups an unpaired, two-tailed, t-test was used. Log-rank (Mantel-Cox) tests were used to determine statistical significance for survival analyses. A p-value significance threshold of P<0.05. Synergy scores were calculated using SynergyFinder (Netphar, University of Helsinki, Helsinki, Finland) with a synergy score greater than 10 indicating a strong synergistic interaction. Graphs were prepared using GraphPad Prism version 10.1.1.

For manuscripts utilizing custom algorithms or software that are central to the research but not yet described in published literature, software must be made available to editors and reviewers. We strongly encourage code deposition in a community repository (e.g. GitHub). See the Nature Portfolio guidelines for submitting code & software for further information.

Data

Policy information about availability of data

All manuscripts must include a data availability statement. This statement should provide the following information, where applicable:
- Accession codes, unique identifiers, or web links for publicly available datasets
- A description of any restrictions on data availability
- For clinical datasets or third party data, please ensure that the statement adheres to our policy

Data supporting the findings of this study are uploaded and available in the Supplementary Information file. Sequencing data generated in this study for patients with informed consent allowing public sharing have been deposited in the dbGaP database under accession code phs003591.v1.p1 [https://www.ncbi.nlm.nih.gov/projects/gap/cgi-bin/study.cgi?study_id=phs003591.v1.p1]. The genomic data are available under restricted access due to patient privacy regulations and institutional ethics policies. Access can be obtained by submitting a data access request through dbGaP, which will be reviewed by the relevant institutional review board. The raw sequencing data are protected and not publicly available due to data privacy laws. The remaining sequencing data for these samples can be accessed upon request from the corresponding author, subject to a completed data transfer agreement. Due to ethical approval restrictions, unrestricted access to the raw data for these samples is not permitted. Mutation frequency analyses were performed using publicly available TCGA HNSCC [https://www.cbioportal.org/study/summary?id=hnsc_tcga] and TCGA CESC [https://www.cbioportal.org/study/summary?id=cesc_tcga] datasets, accessed via cBioPortal. Source data are provided with this paper.

Research involving human participants, their data, or biological material

Policy information about studies with human participants or human data. See also policy information about sex, gender (identity/presentation), and sexual orientation and race, ethnicity and racism.

Reporting on sex and gender	We report biological sex for our cohort of rare tumors in Table 1 of the manuscript. For patients that were prospectively collected and for which informed consent was obtained biological sex is also included in dbGaP upload.
Reporting on race, ethnicity, or other socially relevant groupings	We report race for our cohort of rare tumors in Table 1 of the manuscript. For patients that were prospectively collected and for which informed consent was obtained race is also included in dbGaP upload.
Population characteristics	Not applicable.
Recruitment	For the prospective cohort, patients undergoing surgical biopsy or surgical resection of were recruited. Due to the rare nature of these tumors, for the retrospective FFPE cohort, patients with sinonasal cancer from 2008 to 2020 were identified from FFPE pathology archives at Johns Hopkins. While we sought to identify all patients during that time period, selection bias is possible as some cases may have been missed or FFPE tissue was not available.
Ethics oversight	National Institutes of Health and Johns Hopkins Institutional Review Boards approved this study.

Note that full information on the approval of the study protocol must also be provided in the manuscript.

Field-specific reporting

Please select the one below that is the best fit for your research. If you are not sure, read the appropriate sections before making your selection.

[x] Life sciences [ ] Behavioural & social sciences [ ] Ecological, evolutionary & environmental sciences

For a reference copy of the document with all sections, see nature.com/documents/nr-reporting-summary-flat.pdf

Life sciences study design

All studies must disclose on these points even when the disclosure is negative.

Sample size	As these are rare tumors, there was no pre-determined number of cases, we rather identified and included all possible cases to obtain the highest number of cases feasible.
Data exclusions	No data from patients with HPV-associated or HPV-independent sinonasal squamous cell carcinoma were excluded. The only single data point excluded in our study was one well from Figure 7d, which was excluded from analysis due to a technical issue processing the cell culture plate image. This has been clearly indicated in the Source Data in Figure 7d.
Replication	Cell proliferation and clonogenicity assays were performed 3-4 independent times with similar results observed for each replicate.
Randomization	Not applicable.
Blinding	The bioinformatician performing the sequencing analyses was blinded to patient outcomes. Associations with mutations and patient outcomes were assessed after mutational profiles were determined.

Reporting for specific materials, systems and methods

We require information from authors about some types of materials, experimental systems and methods used in many studies. Here, indicate whether each material, system or method listed is relevant to your study. If you are not sure if a list item applies to your research, read the appropriate section before selecting a response.

Materials & experimental systems

n/a	Involved in the study	
☐	☒	Antibodies
☐	☒	Eukaryotic cell lines
☒	☐	Palaeontology and archaeology
☒	☐	Animals and other organisms
☐	☒	Clinical data
☒	☐	Dual use research of concern
☒	☐	Plants

Methods

n/a	Involved in the study	
☒	☐	ChIP-seq
☒	☐	Flow cytometry
☒	☐	MRI-based neuroimaging

Antibodies

Antibodies used	The patient-derived cell line was characterized by immunofluorescence staining of P63 (SantaCruz, Sc-25268), P40 and cytokeratin AE1/AE3 (SantaCruz, Sc-81714)
Validation	Primary antibodies were validated according to the commercially available manufacturer and our group also validated utilizing negative control tissue when performing immunohistochemistry prior to using in immunofluorescence cell line experiments.

Eukaryotic cell lines

Policy information about cell lines and Sex and Gender in Research

Cell line source(s)	We generated, to our knowledge, the first HPV-associated SNSCC cell line from patient #197, termed NCI-197 cells from a female patient.
Authentication	STR analysis was performed at Johns Hopkins Genetic Resources Core Facility.
Mycoplasma contamination	The Cell line generated tested negative for mycoplasm
Commonly misidentified lines (See ICLAC register)	N/A

Clinical data

Policy information about clinical studies

All manuscripts should comply with the ICMJEguidelines for publication of clinical research and a completedCONSORT checklist must be included with all submissions.

Clinical trial registration	Not applicable
Study protocol	Not a clinical trial
Data collection	Not a clinical trial
Outcomes	Not a clinical trial

Plants

Seed stocks	N/A
Novel plant genotypes	N/A
Authentication	N/A
